# The role of phenotypic switching in the basic biology and pathogenesis of *Candida albicans*

**DOI:** 10.3402/jom.v6.22993

**Published:** 2014-01-15

**Authors:** David R. Soll

**Affiliations:** Developmental Studies Hybridoma Bank, Department of Biology and College of Dentistry, University of Iowa, Iowa City, IA, USA

**Keywords:** white-opaque transition, sexual biofilm, WORI, interacting regulatory network, mating type locus

## Abstract

The “white-opaque” transition in Candida albicans was discovered in 1987. For the next fifteen years, a significant body of knowledge accumulated that included differences between the cell types in gene expression, cellular architecture and virulence in cutaneous and systemic mouse models. However, it was not until 2002 that we began to understand the role of switching in the life history of this pathogen, the role of the mating type locus and the molecular pathways that regulated it. Then in 2006, both the master switch locus WORI and the pheromone-induced white cell biofilm were discovered. Since that year, a number of new observations on the regulation and biology of switching have been made that have significantly increased the perceived complexity of this fascinating phenotypic transition.

‘Phenotypic switching’ in *Candida albicans* was first defined in 1985 as the capacity to undergo spontaneous, reversible transitions between a set number of colony morphologies (1). The definition did not include the bud-hypha transition, which is also considered a form of switching. Variability of colony morphology had been seen in the past in aged colonies or streaks of cells stored on agar at room temperature or in cold rooms, for extended periods of time (2, 3). However, in these earlier studies, reversibility, a trait of phenotypic switching, was never tested. Two papers appeared simultaneously in 1985 which demonstrated that variability of colony morphology included a set number of phenotypes, could be reversible, occurred at high frequency and was stimulated by low doses of UV. One of these systems included switching between seven colony morphologies (4) and another between a smooth and rough colony morphology (5). However, the most interesting story of switching, which has acquired significant attention because of its role in mating, was that of the ‘white–opaque transition’ (6). Having realized that there was more than one type of switching system among natural strains of *C. albicans*, a massive plating experiment was performed by Soll and colleagues (unpublished observations) that included over 100 clinical isolates, in order to identify new switching systems. Several switching systems were identified, including the transition between a hyphal-nonhyphal colony morphology, equivalent to the original smooth–rough transition identified first by Pomes et al. (5), variations of the 3153A switching system identified by Slutsky et al. (4), a petite–large transition and the white–opaque transition (6). Because the white–opaque transition was spontaneous, reversible and between only two phases, but most importantly, because the alternative phenotypes were distinguishable by cellular morphology and vital staining with phloxine B (6, 7), it was selected as the model for studying the role of switching in the life history and pathogenesis of *C. albicans*, and the genes and regulatory networks that controlled the process (1). Between 1987 and 2002, a variety of papers documented the differences in cellular architecture (7–9), phase-specific gene expression (10–16), adhesion and hydrophobicity (17), pathogenicity in mouse systemic and cutaneous models (18, 19), recombination frequencies (20) and surface antigenicity (7–9), between the alternative phenotypes, as well as the role of histone modifications in the regulation of switching in both directions (21, 22). However, during the period preceding 2002, the role of switching and the molecular mechanisms regulating it remained unknown. Thus, the phenomenon was considered interesting, but an enigma.

It was the discovery of the role of white–opaque switching in mating by Miller and Johnson (23) in 2002 that catapulted white–opaque switching to prominence in the community of *C. albicans* researchers. The identification of the mating type locus in 1999 (24) and the demonstration of mating in 2000 (25, 26) preceded this discovery. Miller and Johnson (23) then demonstrated that **a**/α cells of a laboratory strain had to undergo homozygosis at the mating type locus (*MTL*) to switch between white and opaque, and Lockhart et al. (27) showed that this was a general rule for natural *MTL*-homozygous strains. Miller and Johnson (23) also demonstrated that a switch from white to opaque was necessary for mating and this was again found to be a general rule for most natural strains by Lockhart et al. (28, 29). The white–opaque switch, therefore, represented a unique phenotypic transition inserted as a step in the mating program of *C. albicans* and the related species *Candida dubliniensis* (30) and *Candida tropicalis* (31–33). A similar transition was not a requirement for mating amongst members of the *Saccharomyces* clade of the hemiascomycetes, which includes *Saccharomyces cerevisiae* and the pathogen *Candida glabrata*. The discovery that white–opaque switching was a prerequisite for mating in *C. albicans* was followed by the discoveries in 2006 that the transcription factor Wor1, also referred to as Tos9, represented the master switch gene for the white to opaque transition (34–36), and in 2007 that *WOR1* was regulated by the additional transcription factors Czf1, Efg1, and Wor2 (37). As this story continues to emerge, it continues to accumulate new complexity. Rather than review many of the detailed observations that have been covered in prior reviews, especially those pertaining to the molecular regulation of switching and mating (38–44), an attempt will be made here to provide more of an overview of how the rapidly accumulating observations have changed our perception of switching in the last decade. Special consideration will be given to the role that *MTL*-homozygous biofilm formation may play in the life history of *C. albicans* and why it may be the key to understanding why the white–opaque transition originally evolved.

## What is the relationship between switching and mating?

**Fig 1:**
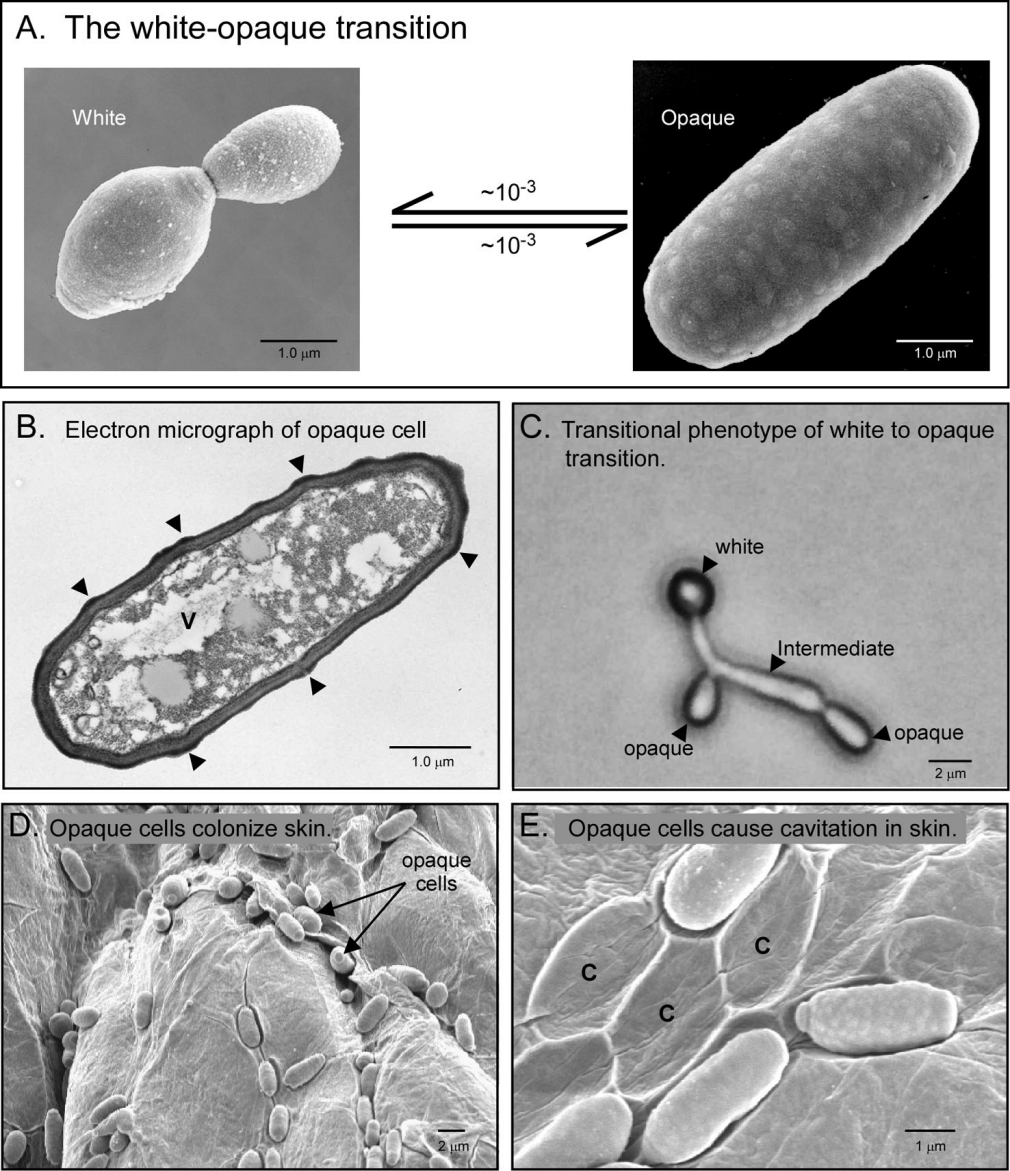
Scanning electron micrographs of white–opaque switching, which involves a dramatic change in cellular phenotype and differences in the capacity to colonize skin. *A*. The transition is between the white (**a/a** or α/α) budding yeast cell and a unique oblong, pimpled opaque (**a/a**, α/α) cell. The frequencies given vary according to the method used to measure them, the strains used, environmental conditions, etc. *B*. Transmission electron micrograph of an opaque cell reveals wall pimples and a giant vacuole. Arrow heads indicate pimples; V indicates a large vacuole. *C*. The transition from the white budding cell to oblong opaque cell involves an elongate intermediate phenotype. *D*. Opaque cells, but not white cells, readily colonize the skin of a new-born mouse, in many cases sinking into an induced cavity. *E*. Removal of opaque cells from skin reveals cavities (C) formed under them in the skin cells.

The vast majority of natural strains of *C. albicans*, a predominately diploid organism, are heterozygous at the mating type locus (27, 45–48). To mate, diploid cells must undergo homozygosis at the mating type locus to **a/a** or α/α by either gene conversion, crossing over or the loss of one copy of chromosome 5, followed by duplication of the retained copy (49, 50). Then the resulting *MTL*-homozygous cell must switch (23, 28) from a ‘white’ **a/a** or α/α cell phenotype, architecturally similar to the **a**/α yeast cell phenotype, to the unique oblong opaque cell phenotype, with a giant vacuole and a cell wall containing pimple-like structures (Fig. 1A and 1B) (6, 7). The subsequent mating process that takes place between opaque **a/a** and α/α cells is then cytologically similar, but not identical, to that of the highly studied species *Saccharomyces cerevisiae*. Alternative mating types secrete pheromones that stimulate cells of opposite mating type to evaginate (29, 51–53). The evaginations elongate and fuse and the nuclei migrate into the conjugation tube (28, 54). The nuclei in turn fuse (54). A daughter cell forms at the position of the fused, tetraploid nucleus (29, 54). These events are similar to those in the mating process of haploid **a** and α *S. cerevisiae* cells. However, unlike the short mating evaginations formed by *S. cerevisiae*, *C. albicans* is capable of forming long mating tubes (27, 28, 51, 52, 54) that can grow up to several cell diameters in length (55), suggesting that *C. albicans* mating tubes formed in the host may have to undergo chemotropism over long distances to fuse. Second, instead of forming a diploid daughter cell after **a** and α cell fusion, as is the case for *S. cerevisiae*, *C. albicans* generates a tetraploid (**a/a**/α/α). However, unlike the diploid fusion product of *S. cerevisiae*, which undergoes meiosis to return to the haploid state, the tetraploid fusion product of *C. albicans* returns to the diploid state by random loss of chromosomes (56), which involves recombination and requires Spo11, a protein involved in meiosis in other fungi (57). Hickman et al. (45) recently demonstrated that diploid strains can give rise to rare haploid strains that must still undergo the white–opaque transition to mate. They suggested that chromosome loss leading to haploids may rid cells of recessive lethal alleles. Alby et al. (58) also showed that a unisexual population, in the absence of the extracellular protease that breaks down pheromone (i.e. in the *bar1* ^*-*^ mutants), can undergo low levels of mating through an autocrine-like system.

The discovery that *MTL*-haploid as well as *MTL*-homozygous diploid strains normally must undergo a switch from white to opaque to mate, reinforced the importance of the switching prerequisite for mating in *C. albicans*. But none of these observations explained why *C. albicans* and related species in the *Candida* clade of the hemiascomycetes, have added to their mating process so extravagant a differentiation as white–opaque switching, while members of the *Saccharomyces* clade have not. Why not mate immediately when a cell becomes homozygous at the *MTL* locus, as is the case for haploid *S. cerevisiae*? Why undergo such a massive change in gene expression, which appears to involve a unique and complex regulatory circuit? Why produce two phenotypes? And why generate such a unique opaque phenotype (Fig. 1A and 1B) (7)? The answer may be, at least in part, related to unique host–pathogen interactions and the role played by pheromone-induced white cell biofilm formation in the opaque cell mating process in the host (59).

## Is white cell biofilm formation the reason for switching?

**Fig 2:**
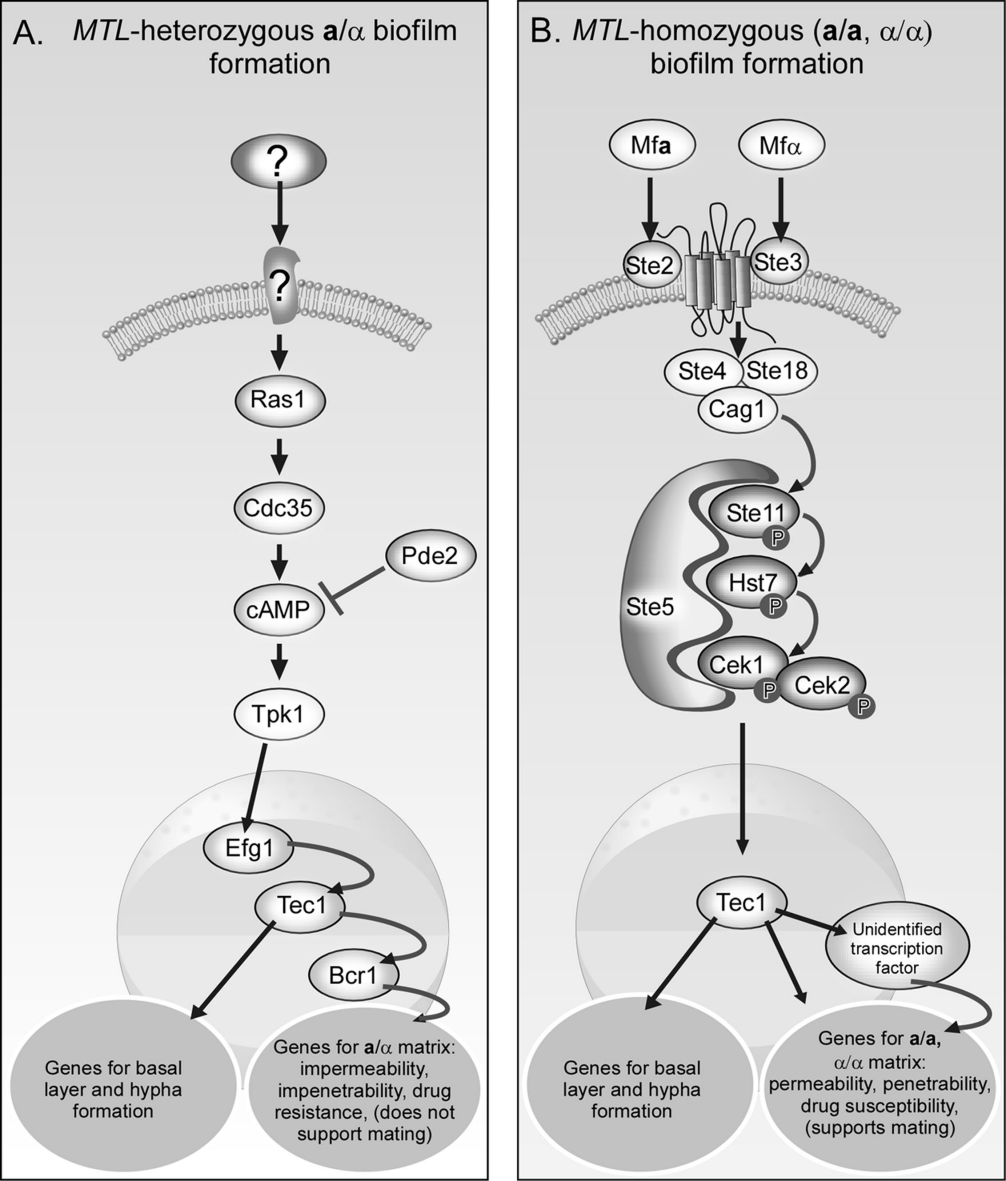
While formation of a ‘pathogenic’ *MTL*-heterozygous (**a**/α) biofilm is regulated by the Ras1/cAMP pathway, formation of a sexual *MTL*-homozygous biofilm is regulated by the MAP kinase pathway (72). Regulation in this case was analyzed in **a**/α, and alternatively in **a/a** or α/α biofilms, formed on silicone elastomer for 48 hours at 25°C in air, in RPMI 1640 medium. Note that even though the final biofilms are architecturally similar, the characteristics resulting from different matrices differ.

When *MTL*-homozygous cells switch from white to opaque, they become mating-competent (23, 27). Opaque **a/a** cells secrete **a**-pheromone that induces the α/α cell mating response and α/α cells secrete α-pheromone that induces **a/a** cells to undergo a similar mating response (29, 51–53). As in *S. cerevisiae* (60, 61), gradients of the two pheromones then direct chemotropism (62), leading to fusion at the tips of the conjugation tubes of alternative mating types. Because white cells are mating-incompetent, they do not undergo a mating response when treated with pheromones of opposite mating type, but Lockhart et al. (29) initially reported that they still responded to pheromone by up-regulating several genes involved in the pheromone signal transduction pathway. Pheromone receptors were also demonstrated on the surface of white cells (62). It was subsequently demonstrated that α-pheromone stimulated adhesion in white **a/a** cell populations (62–64) and that minority opaque α/α cells, a presumed source of pheromone, when seeded in a majority white **a/a** cell population developing a conventional biofilm on silicone elastomer in RPMI 1,640 medium (65), increased the thickness of the white cell biofilm (64, 66). It was further demonstrated that homogeneous populations of white **a/a** cells to which no minority opaque cells were added, autostimulated biofilm formation by low level spontaneous switching to opaque **a/a** cells that in turn secreted α-pheromone, in an unorthodox paracrine system (67). Mutational analyses revealed that the pathway for the pheromone-induced mating response in *C. albicans* was conserved in the hemiascomycetes and hence was the same pathway as the pheromone-induced mating response of *S. cerevisiae* (i.e. all of the tested components of the *C. albicans* mating response pathway were orthologs of the *S. cerevisiae* mating response pathway) (64, 68–73). The major regulatory pathway included pheromones (Mfα, Mf**a**), the pheromone receptors (Ste2, Ste3), the trimeric G protein complex (Cag1, Ste4, Ste18), the MAP kinase cascade (Ste11, Hst7, Cek1, Cek2), the Ste5 scaffold and the targeted transcription factor (Cph1). Surprisingly, mutational analyses revealed that the pathway up to, but not including, the transcription factor for white cell biofilm formation on silicone elastomer in RPMI 1,640 medium, was the same as the mating pheromone response pathway (Fig. 2B) (64, 66). The transcription factor for white cells forming a conventional biofilm (65) on silicone elastomers in RPMI 1,640 medium was first identified as Tec1, one of the transcription targets for **a**/α biofilm formation (74), which in **a**/α cells is regulated by the Ras1/cAMP pathway. Cph1 was found to play a role, but a minor one, for white cells forming a biofilm in RPMI 1,640 medium (64). Although this result was recently challenged by Lin et al. (63), who presented evidence that the main targeted transcription factor for the white cell biofilm response was Cph1 and that Tec1 played a minor role, results opposite those of Sahni et al. (74), the cell preparations analyzed by Lin et al. (63) were incubated in Lee's medium rather than RPMI 1,640, on plastic rather than elastomer, and developed for 24 hours rather than 48 hours. The conditions Lin et al. (63) employed were those used in the original adhesion assay, not the biofilm assay, of Sahni et al. (74). Daniels et al. (65) recently reported that **a/a** biofilms formed in air in Lee's medium, on plastic or elastomer, for 24 hours, consisted of a yeast cell polylayer with no upper region of vertical hyphae embedded in a dense matrix, the latter a major characteristic of conventional *C. albicans* biofilms. These conflicting results continued to be actively investigated when this review was in preparation, but the outcome will have little bearing on the main story that follows.

It has been proposed that the formation of white cell biofilms in response to pheromone released by minority opaque cells, may explain why the white–opaque transition evolved (44, 59, 75). In 2006, Daniels et al. (62) demonstrated that when minority, mating-competent **a/a** and α/α cells were seeded in majority white **a/a–**α/α cell biofilms, they underwent chemotropism. They therefore hypothesized that white cell biofilms formed to facilitate mating of minority opaque cells, which arise in white cell populations by spontaneous switching (6, 7, 76, 77). Park et al. (78), using a complementation strategy recently demonstrated that white **a/a** and α/α biofilms facilitated mating between seeded minority opaque **a/a** and α/α cells at frequencies 10 to over 100 times that obtained in **a**/α biofilms. This led to the hypothesis that even though the general architecture of *MTL*-homozygous biofilms was similar to that of *MTL*-heterozygous biofilms, the matrices were different, and only the **a/a** and α/α matrices supported mating. Functional assays were consistent with this hypothesis. While *MTL*-heterozygous **a**/α biofilms were highly impermeable to low and high molecular weight molecules, highly impenetrable by human polymorphonuclear leukocytes (PMNs) and highly resistant to fluconazole, *MTL*-homozygous (**a/a**, α/α or mixed **a/a**–α/α) biofilms were permeable, penetrable and fluconazole-susceptible (64, 66, 67, 79). It was therefore proposed that while *MTL*-heterozygous biofilms represented ‘pathogenic biofilms’, *MTL*-homozygous biofilms represented ‘sexual biofilms’.

It was suggested that the functional distinction between *MTL*-heterozygous and *MTL*-homozygous biofilms may be related to the differences in matrix (44, 59, 74, 75). Both *MTL*-heterozygous and *MTL*-homozygous biofilms that form on silicone elastomer in RPMI-1,640 medium after 48 hours in air or in 20% CO_2_ at 37°C, consist of a basal polylayer of yeast cells and a thick upper layer of vertically oriented hyphae embedded in a matrix (62, 64, 65, 73, 75). The upper hyphae-matrix region represents 70–80% of the volume of a biofilm formed on silicone elastomer in RPMI 1,640 medium in air or 20% CO_2_, at 37°C (65). While the formation of the basal layer and formation of long hyphae in the upper layer of both *MTL*-heterozygous and *MTL*-homozygous biofilms appear to be regulated by the transcription factor Tec1, the matrix of the former appear to be regulated primarily by Bcr1 (73, 79–81), while that of **a/a** and α/α biofilms is regulated by a still unidentified transcription factor (Fig. 2A and 2B) (73, 79). Bcr1 has been shown to regulate a variety of genes in **a**/α biofilms involved in impermeability and impenetrability (79). Hence it was proposed that the *MTL*-heterozygous biofilm matrix may have evolved to resist penetration by antibodies and phagocytic cells, but these characteristics proved incompatible with chemotropism in the mating process. For the white *MTL*-homozygous biofilm matrix to have evolved to facilitate chemotropism and mating between minority opaque **a/a** and α/α cells, it would have to be permeable enough to allow gradients of pheromone to form for chemotropism and penetrable enough to allow conjugation tubes to move readily up these gradients. Hence **a/a** and α/α biofilms must be permeable and penetrable. And indeed Park et al. (78) have shown that seeded minority opaque cells form longer mating tubes and mate at far higher frequencies in *MTL*-homozygous biofilms than *MTL*-heterozygous biofilms. While this hypothesis makes sense, the proof for it lies exclusively in the results from *in vitro* models of biofilm formation. It still remains to be demonstrated that it reflects natural situations in the host.

## How is switching regulated?

Slutsky et al. (6) discovered the white–opaque transition in an isolate from a blood stream infection, obtained from an immunosuppressed patient at the University of Iowa Hospitals and Clinics in 1986, but it was not until the early 2000s that any observations pertaining to regulation emerged. During the intervening 15-year period, reversible genetic mechanisms as well as epigenetic mechanisms were considered equally, as candidates for the regulation of switching. In 2001, it was first demonstrated that an inhibitor that targeted the histone deacetylase Hda1 and deletion of the histone deacetylase gene *HDA1* promoted the transition from white to opaque (21), and then that deletion of the deacetylase gene *RPD3* resulted in a decrease in the frequency of switching in both the white to opaque and opaque to white direction (22). These results indicated that transitions in chromatin state, and hence the activation–deactivation of a particular gene, or sets of genes, through chromatin modification might be the basis of reversible switching. In 2002, Lan et al. (10), using cDNA microarrays, showed that roughly 5% of the identified genes in the *C. albicans* genome were up or down regulated, to different degrees, in the transition, and that the patterns suggested fundamental changes in metabolism. The list of regulated genes was reassessed by Tsong et al. (82) in 2003, verifying that approximately 400 genes were regulated in the transition. But it was in 2006 that a potential ‘master switch gene’ was discovered. In that year, three laboratories simultaneously identified, by different approaches, the master switch locus *WOR1* (also referred to as *TOS9*), which encoded a transcription factor (34–36). Deletion of *WOR1* blocked the spontaneous transition from white to opaque, indicating that the white phase was the default phenotype. Evidence provided primarily by Zordan et al. (36) suggested that high levels of Wor1 induced the transition from white to opaque. Zordan et al. (37) subsequently showed that Wor1 was regulated by additional genes in an interacting network of activation and repression that included autoregulation, by all of the identified regulatory components, which included Wor1. The initial transcription factors found to regulate *WOR1* included Czf1, Efg1 and Wor2 (37). Subsequently it was discovered that additional regulators of Wor1, Ahr1 and Wor3, were involved in controlling expression of the alternative phenotypes, resulting in a minimum of six regulators in the interacting networks (83). This led to a model in which expression of Efg1 supported the white phenotype, while expression of Wor1 supported the opaque phenotype, and that each of these were in turn regulated by an alternative interacting regulatory network in the two cell phenotypes. The network components, Czf1 and Ahr1 regulated Efg1 in the white phase, and Efg1, Czf1, Ahr1, Wor2 and Wor3 regulated Wor1 in the opaque phase (83). ChIP-chip analyses indicated binding of each factor with the promoters of the other factors, as well as to their own promoters (36, 37, 83). In 2010, Tuch et al. (84) found, using RNA-sequencing, that three times as many transcripts were regulated by switching than was previously reported, many non-coding sequences and many encoding short proteins. Some of the non-coding regions appeared to encode antisense sequences to mRNAs. Lohse et al. (85) then demonstrated that a 300 amino acid region of Wor1 represented a unique binding region, a WOPR box. This regulatory sequence was conserved among the fungi, and in *Histoplasma capsulatum*, it was involved in the binding of Ryp1, a regulator of the yeast–mycelium transition (86). In addition, Wang et al. (87) showed that Zcf37, a zinc finger protein, stabilized the white phenotype. These increasingly complex regulatory networks and individual regulatory components presumably are influenced by environmental cues or specific genetic configurations, such as that of the mating type locus. The models for these networks were based on elegant binding data and expression profiles, and in some cases await verification through functional analyses in which a series of deletion derivatives of each promoter are generated in frame with a reporter gene. The 2,300 bases immediately upstream of *EFG1* had been functionally characterized in this way by generating 22 promoter deletion derivatives fused to the reporter gene *RLUC* (88). Unfortunately, this study covered only one eighth of the intergenic region between the closest upstream gene and the open reading frame of *EFG1*, an intervening region composed of 10,000 base pairs. Even so, one discrete cis acting activation sequence was identified for white phase expression, but no discrete cis acting activation sequences were observed for opaque phase expression. Srikantha et al. (89) also found that the transcription start sites differed for white and opaque phase expression, suggesting overlapping white and opaque *EFG1* promoters, and demonstrated differences in the 3' ends of the alternative transcripts. Hence, regulation of the components of the transcription factor networks regulating *WOR1* and *EFG1* appear to be complex, and may involve a variety of additional genes, results consistent with those of Tuch et al. (84).

And as noted, in addition to transcription factors, general regulators of chromatin structure through histone and DNA modification have been implicated in the regulation of switching. Programmed changes in chromatin structure increase the complexity of the models of regulation, as first noted by Hnisz et al. (90). In addition to the initial observation that the histone deactylases Hda1 and Rpd3 played a role in switching frequencies (21, 22), Hnisz et al. (90) showed that the Set3/Hos2 histone deactylase complex regulated switching, depending on the methylation status of H3. Tscherner et al. (91) showed that the acetyltransferase Hat1 was involved in the white to opaque transition, and Stevenson and Liu (92) showed that the acetyltransferase Rtt109 and the deactylase Hst3 were also involved in switching and that nucleosome assembly factors Caf-1 and Hir were involved (93). Interestingly, they showed that overexpression of *WOR1* could override some of the mutant defects, suggesting that the chromatin effectors target the *WOR1* locus. And finally Mishra et al. (94) have presented evidence that methylation of genes regulated by switching differ between the white and opaque phase. These results provide a second level of complexity to the binding networks of transcription factors regulating *WOR1* and *EFG1* expression in the white–opaque transition.

And in the face of such complexity at the level of gene regulation, it seems likely that different components of the networks are influenced by the environment. Indeed, Tong et al. (95) recently found that deletion of *WOR2* did not block switching from white to opaque when cells were induced to undergo the transition by substituting glucose with GlucNAc in Lee's medium in 5% CO_2_. Hence, a change in environmental conditions could obviate a major component of the network regulating activation of *WOR1*. Moreover, regulation of switching may not solely be a function of the expression levels of the transcription factors. Indeed it may involve modification of such factors, most notably through kinases and phosphatases. Srikantha et al. (35) identified a putative protein kinase A (PKA) binding site consensus sequence encoded in the region surrounding base pair 190 of the open reading frame of *WOR1*. If all of the transcription factors were in turn regulated by secondary modifications, one wonders how complex the regulatory networks could be, when expanded to include modification pathways. The sheer complexity of the networks now emerging argues that switching was not simply an evolutionary event based on a single genetic alteration, but rather a complex evolutionary process involving the recruitment of hundreds of genes into complex, interacting networks, in order to undergo a complex differentiation to a new cell type and to undergo opaque-induced or autostimulated white cell biofilm formation.

**Fig 3:**
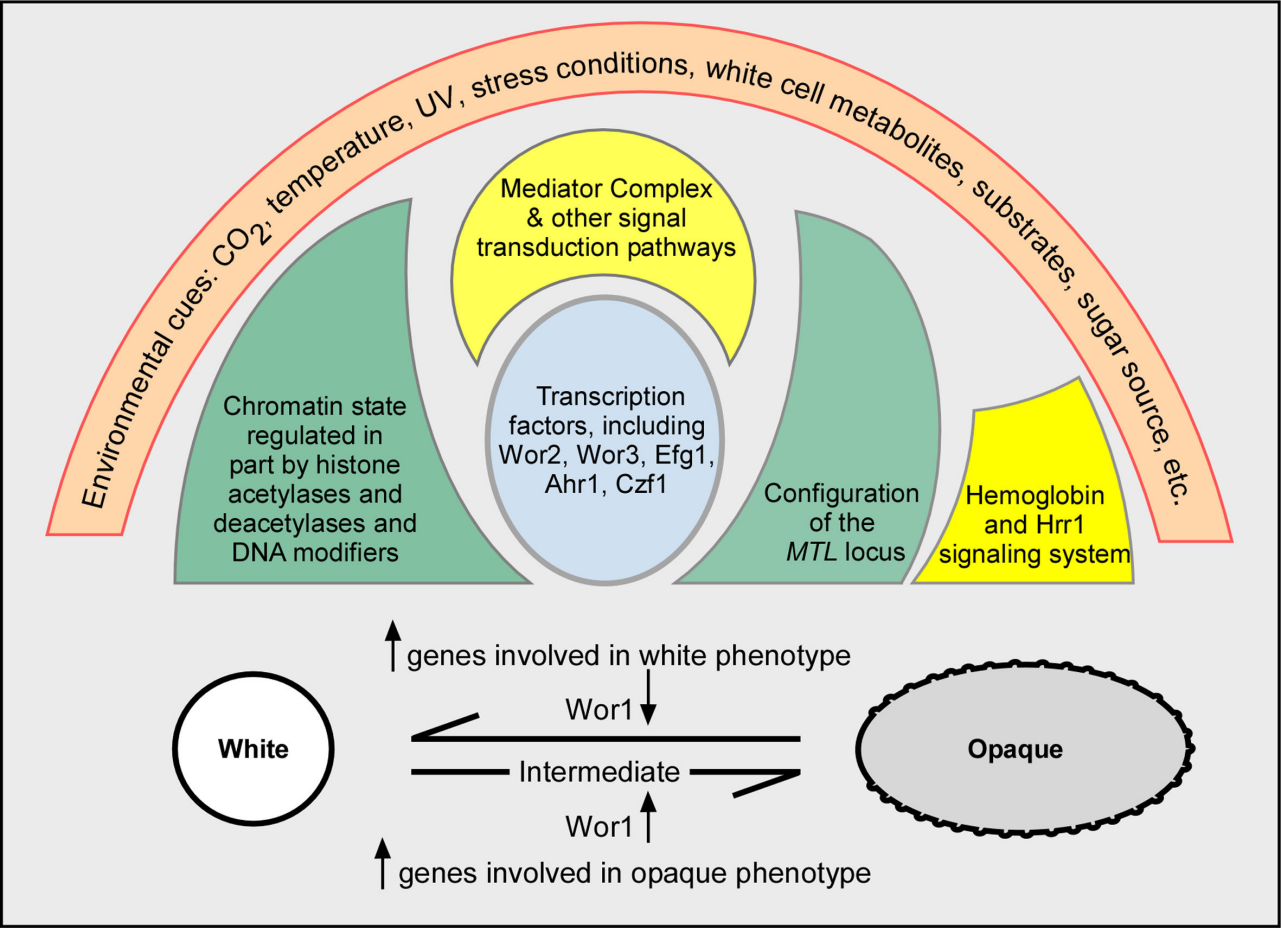
The evolving complexity of the regulation of the white–opaque transition. The arrows denote upregulation (↑) or down regulation (↓).

In a more inclusive description presented in Fig. 3, I have layered the different aspects of regulation as shells impacting Wor1, which may still prove to be the central off (white)–on (opaque) regulatory switch. As I have already alluded to, molecules regulating the general state of chromatin, transcription factor networks, secondary modifiers of transcription factors, signal transduction pathways regulating these changes, the genes encoding the proteins directly involved in the mechanics of switching and finally, the configuration of the *MTL* locus, all appear to play roles in regulating the white–opaque transition. And superimposed on all of these layers of regulation are the various characteristics of the environment (i.e. the signals) and the genetic backgrounds of strains other than that of the *MTL* locus. As discussed in the next section, some environmental signals can override the exclusivity of switching in an *MTL*-homozygous background, and, as already noted, the exclusivity of the components of the established networks regulating *WOR1*. Environmental conditions that can influence the frequency of switching and the stability of the opaque phenotype include the level of CO_2_ in the environment (96, 97), the sugar source (95, 98), temperature (6, 14, 77), low doses of UV irradiation (99), genotoxic and oxidative stress (100), and *in vitro* oxidants and white blood cell metabolites (101).

Finally, a caveat is missing from all recent studies of regulation. While the opaque cell can immediately form a white daughter cell in the opaque to white transition, white cells appear to undergo a developmental transition to the opaque phenotype through an intermediate phenotype, at least when incubated as single cells on an agar cushion (Fig. 1C) (76). This transition has not been studied at the molecular level, suggesting that the regulation of *WOR1* may be even more complex, given it may include complex multiphenotypic developmental transitions in one direction. And that temporal complexity may involve incremental changes at the regulatory level in the opaque to white direction. This kind of analysis has been performed in the white to opaque direction. A study by Lohse and Johnson (102) has revealed that opaque cells change to a white regulatory signature before they commit to the white cellular phenotype. And to make the story even more complex, Zhang et al. (103) recently demonstrated that a conserved mediator complex, which has been shown in animal cells to transmit regulatory signals to transcription machinery, plays a fundamental role in regulating the white–opaque transition in *C. albicans*. Deletion of the Med3 subunit of the large complex destabilizes switching, even with a fully operational Wor1-based circuit, affecting the frequency of the white to opaque transition and blocking the capacity to mate.

## Switching in a/α cells

Originally, it was believed that switching was a characteristic only of *MTL*-homozygous cells (23, 27). However, present descriptions of the regulation of the white–opaque transition rarely include the role of the **a**1–α2 co-repressor complex. Miller and Johnson (23) first showed that this complex suppressed switching by deleting **a**1 or α2, and Lockhart et al. (27) subsequently showed it to be the case for most natural strains by correlating switching with *MTL* homozygosity. Soon afterwards, Pendrak et al. (104) showed that the hemoglobin response gene (*HBR1*) is in a signaling pathway that regulates switching. They concluded at that time that it affected switching through regulation of the **a**1–α2 co-repressor complex. They were able to delete this gene and show that deletion down-regulated *MTL* **a**1, leading to a switch to the opaque phenotype in an **a**/α strain. More recently, Xie et al. (105) showed that at physiological levels of CO_2_ (5%) with GlcNAc, rather than glucose, as the sole carbon source, select clinical **a**/α strains underwent white–opaque switching without undergoing *MTL*-homozygosis. Hence, the idea that white–opaque switching was restricted to *MTL*-homozygous cells has to carry the restriction ‘under select conditions’ – i.e. in air and with a sugar source other than GlcNAc. Moreover, in a study by Pande et al. (106), it was demonstrated that **a**/α cells injected into the gastrointestinal tract of a mouse exhibited a cell morphology that was elongate like opaque cells, but lacked opaque cell pimples. This opaque-like **a**/α cell expressed *WOR1* at elevated levels, raising the question of what other roles are played by the opaque phenotype or portions of it. Previously, Ramirez-Zavala et al. (97) found that passing *MTL*-homozygous white cells through the gastrointestinal tract of a mouse resulted in increased levels of switching to the opaque phase, adding weight to the notion that phenotypic characteristics shared by the opaque-like, *MTL*-heterozygous cell (106) and the opaque *MTL*-homozygous opaque cells (97) may provide adaptive value for colonizing the gastrointestinal tract of mammals.

## What then does switching have to do with commensalism and pathogenesis?

The white–opaque transition, therefore, may have evolved not only to facilitate mating, an apparently rare but important event in the life history of *C. albicans*, but also to host colonization. A number of additional, intriguing aspects of white and opaque cells related to commensalism and pathogenesis bare on this point. First, although white cells are more virulent than opaque cells in a systemic mouse model (19), the reverse is true in a cutaneous model of infection (18). Opaque cells more readily colonize skin (Fig. 1D) than white cells, causing cavities in the skin surface (Fig. 1E) (18). Misexpression in white cells of *SAP1*, an aspartyl protease gene up-regulated in opaque cells (12, 107, 108), confers in white cells the capacity to colonize skin and cause cavitation (18), perhaps by exposing adhesins and weakening the cortex of skin cells. But perhaps what may be the most intriguing phenotypic characteristic of opaque cells, is that they lose the capacity to release a potent chemoattractant for PMNs (109). This attractant is released only by white cells. In practical terms, the lack of attractant could make opaque cells, but not white cells, invisible to PMNs. This may represent a way of protecting mating-competent cells from phagocytosis. Sasse et al. (110) further demonstrated that when opaque and white cells were mixed together with human PMNs, the PMNs only engulfed white cells, even though they physically encountered opaque cells. And Lohse and Johnson (111) demonstrated that both mouse and *Drosophila* phagocytic cells had a preference for white cells over opaque cells.

And let us not forget the biofilm story (59, 75). If white **a/a** and α/α cell biofilms are there to protect mating, why are they so much more permeable to low and high molecular weight molecules, so much more penetrable by human PMNs and so much more susceptible to antifungal agents than **a**/α biofilms? Could it be, as suggested, that forming a sexual biofilm requires a matrix that lacks pathogenic traits, because these traits are incompatible with mating (59, 75)? While the explanations for the roles of white–opaque switching in commensalism and pathogenesis are quite interesting, they will remain speculative until we demonstrate they play such roles during commensalism and pathogenesis in humans. Models in which the initial inocula are large and introduced in a very short time frame through an unnatural portal such as injection into the mouse tail vein, may not reflect an *in vivo* scenario. And the use of inbred or immunosuppressed mice may complicate interpretations related to the human commensal state. It therefore seems appropriate to argue that it is time to test whether the phenotypic, developmental, metabolic and regulatory states of *C. albicans* and related species, including white–opaque switching, that have been elucidated and characterized *in vitro* and to a lesser extent in animal models, are valid representations of events in the human host. At a minimum, *in vitro* conditions should be altered to mimic more accurately conditions in the human host.

## Concluding comments

**Fig 4:**
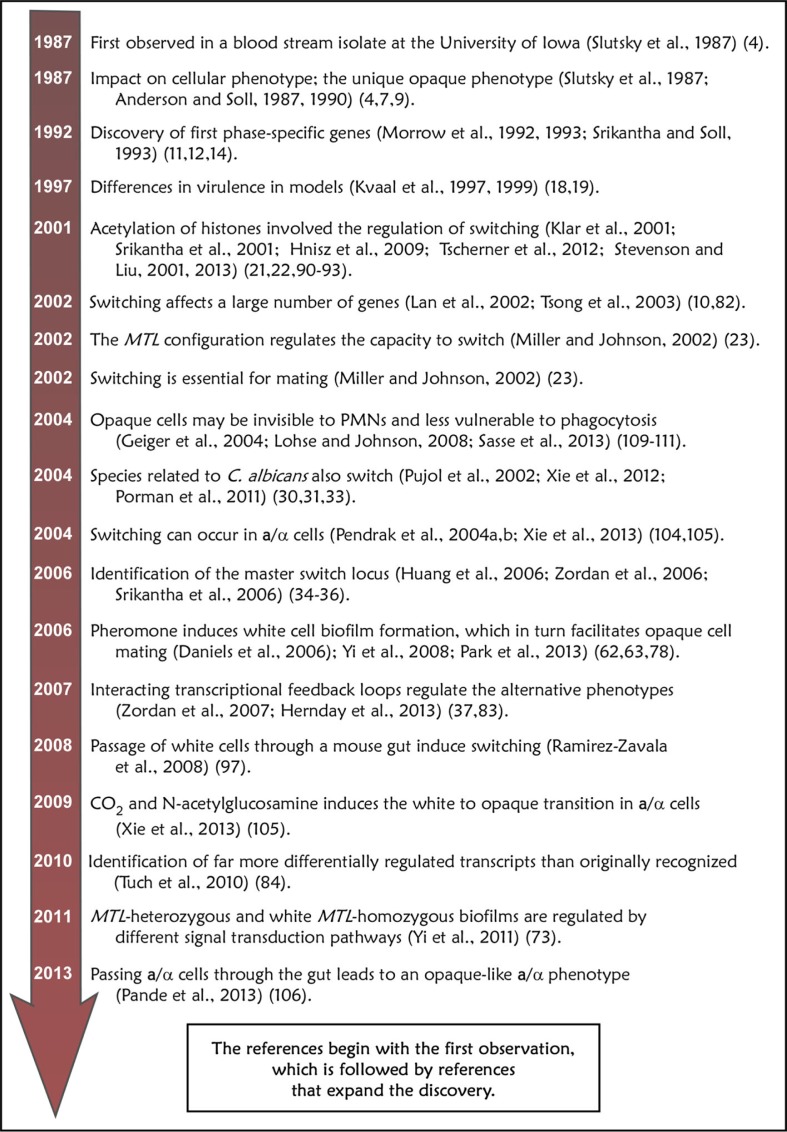
Landmark discoveries directly related to the white–opaque transition in *C. albicans*. This represents an incomplete list based on this author's view of the field.

At this writing, well over 130 articles have been published on various discoveries related directly to the white–opaque transition since its discovery 26 years ago (6). What may be deemed some of the landmark discoveries and subsequent studies that expanded or modified them, are presented in Fig. 4. It is clear that the discovery of switching ignited interest in scientists with a variety of expertise. Interest in switching continues to grow rapidly, as evidenced by the fact that well over 15% of the articles directly pertaining to the white–opaque transition, written since 1987 were published in the first 9 months of 2013. Thus, this story is far from over.
